# Di-*n*-but­yl[*N*′-(3-meth­oxy-2-oxidobenzyl­idene)-*N*-phenyl­carbamohydrazono­thio­ato]tin(IV): crystal structure, Hirshfeld surface analysis and computational study

**DOI:** 10.1107/S2056989021001870

**Published:** 2021-02-23

**Authors:** Enis Nadia Md Yusof, Huey Chong Kwong, Thiruventhan Karunakaran, Thahira B. S. A. Ravoof, Edward R. T. Tiekink

**Affiliations:** a Chemistry Section, School of Distance Education, Universiti Sains Malaysia, 11800 USM, Pulau Pinang, Malaysia; b Department of Chemistry, Faculty of Science, Universiti Putra Malaysia, UPM Serdang 43400, Malaysia; c Research Centre for Crystalline Materials, School of Medical and Life Sciences, Sunway University, 47500 Bandar Sunway, Selangor Darul Ehsan, Malaysia; d Centre for Drug Research, Universiti Sains Malaysia, 11800 Minden, Pulau Pinang, Malaysia; e School of Chemical Sciences, Universiti Sains Malaysia, 11800 USM, Pulau Pinang, Malaysia; f Foundry of Reticular Materials for Sustainability (FORMS), Materials Synthesis and Characterization Laboratory, Institute of Advanced Technology, Universiti Putra Malaysia, 43400 Serdang, Selangor Darul, Ehsan, Malaysia

**Keywords:** crystal structure, organotin, Schiff base, thio­semicarbazone, hydrogen bonding, Hirshfeld surface analysis

## Abstract

The title diorganotin Schiff base derivative, [Sn(C_4_H_9_)_2_(C_15_H_13_N_3_O_2_S)], features a penta-coordinated tin centre defined by the N,*O*,*S*-donor atoms of the di-anionic Schiff base ligand and two methyl­ene-C atoms of the *n*-butyl substituents. The resultant C_2_NOS donor set defines a geometry inter­mediate between trigonal–bipyramidal and square-pyramidal. In the crystal, amine-N—H⋯O(meth­oxy) hydrogen bonding is found in a helical, supra­molecular chain propagating along the *b*-axis direction. The chains are assembled into a layer parallel to (01) with methyl­ene-C—H⋯π(phen­yl) inter­actions prominent; layers stack without directional inter­actions between them. The analysis of the calculated Hirshfeld surface showed the presence of weak methyl­ene-C—H⋯π(phen­yl) inter­actions and short H⋯H contacts in the inter-layer region. Consistent with the nature of the identified contacts, the stabilization of the crystal is dominated by the dispersion energy term.

## Abstract 2

The C_2_NOS donor set about the tin atom in the title compound has a geometry inter­mediate between trigonal–bipyramidal and square-pyramidal. In the crystal, a helical, supra­molecular chain along the *b*-axis direction features amine-N—H⋯O(meth­oxy) hydrogen bonding.

## Chemical context   

Thio­semicarbazones are an important class of compounds that have received wide attention due to their many biological and pharmacological properties, such as anti-bacterial, anti-viral, anti-neoplastic and anti-malarial activities (Kovala-Demerzi *et al.*, 1997 ▸; Hu *et al.*, 2006 ▸; Khan & Yusuf, 2009 ▸). Thio­semicarbazone Schiff bases are similar to their di­thio­carbazate counterparts in that complexation with a metal centre is achieved *via* the nitro­gen and sulfur atoms following deprotonation of the S—H and N—H groups (Đilović *et al.*, 2008 ▸; Wiecek *et al.*, 2009 ▸; Pavan *et al.*, 2010 ▸; Parrilha *et al.*, 2011 ▸; Singh *et al.*, 2016 ▸; Palanimuthu *et al.*, 2017 ▸). Tin(IV) compounds of 3-meth­oxy­salicyl­aldehyde thio­semicarbazone have been evaluated for their *in vitro* cytotoxicity against a line of human T lymphocyte cells, Jurkat cells (Khandani *et al.*, 2013 ▸): in this study, a structure–activity analysis for the di­alkyl­tin(IV) compounds indicated that cytotoxicity increased with the length of the alkyl carbon chain of the tin-bound substituents. Thus, the cytotoxicity was in the order of dibutyl > diphenyl > dimethyl (Khandani *et al.*, 2013 ▸). The ability of the 2-acetyl­pyridine *N*(4)-cyclo­hexyl­thio­semicarbazone Schiff base (*L*H_2_) and its distorted penta­gonal bipyramidal tin(IV) compound, [Ph_2_Sn(*L*)(OAc)]·EtOH, to inhibit tumour cell growth against HepG2 cells has also been reported (Liu *et al.*, 2017 ▸). This study showed the tin(IV) compound to exhibit threefold higher cytotoxic potency compared to the free ligand, *i.e*. with IC_50_ values of 3.32±0.52 and 10.10±2.07 µ*M*, respectively, and to be more potent than the reference drug mitoxantone (IC_50_ = 5.3±2.38 µ*M*). Significant activity was also observed in an *in vitro* cytotoxic assay of tin(IV) compounds of 2-hy­droxy-5-meth­oxy­benzaldehyde-*N*(4)-meth­yl­thio­semicarbazone (Salam *et al.*, 2016 ▸), di­phenyl­tin(IV) compounds of 2-benzoyl­pyridine *N*(4)-phenyl thio­semi­carbazone and 2-acetyl­pyrazine *N*(4)-phenyl­thio­semi­carbazone (Li *et al.*, 2011 ▸) in comparison to the standard drugs used. It may be concluded that the coordination of the Schiff base ligand to the tin(IV) centre enhanced cytotoxic activity, where the reported IC_50_ values were better than standard drugs.
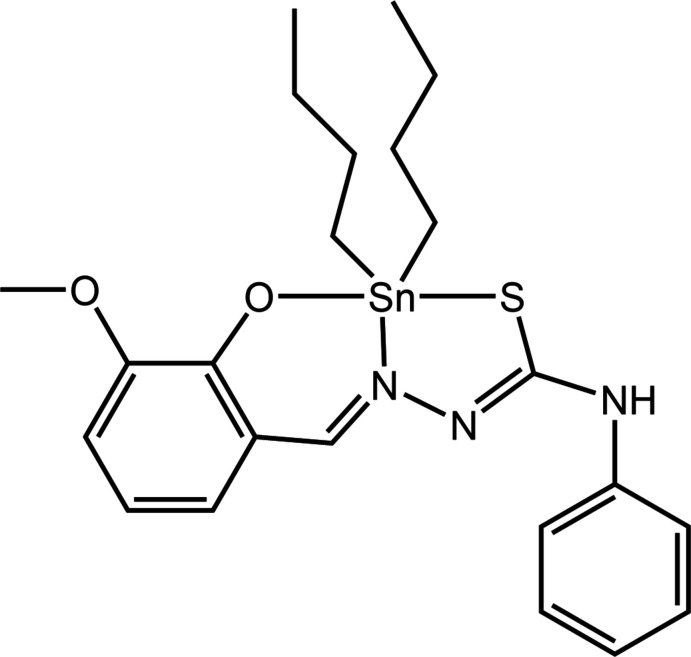


Further, the enhancement of cytotoxicity in the di­phenyl­tin derivatives has been attributed to the presence of these phenyl groups, which suggested inter­actions between the tin-bound phenyl groups with intra-cellular biomacromolecules. An independent biological study suggested that the diffusion, lipophilic character and steric effects associated with the ligand could also be factors in determining cytotoxic activity (Salam *et al.*, 2016 ▸). The improvement of cytotoxic activity was also suggested to be due to the presence of OH/NH groups, which enabled hydrogen bonding with DNA base pairs (Haque *et al.*, 2015 ▸). As part of our on-going studies in the structural elucidation and cytotoxic activity of tin(IV) compounds containing thio­semicarbazones Schiff base (Yusof *et al.*, 2020 ▸), herein are described the synthesis of the title di­butyl­tin(IV) derivative, (I), its single crystal X-ray diffraction analysis and a detailed study of supra­molecular association by an analysis of the calculated Hirshfeld surfaces and computational chemistry.

## Structural commentary   

**Figure 1:**
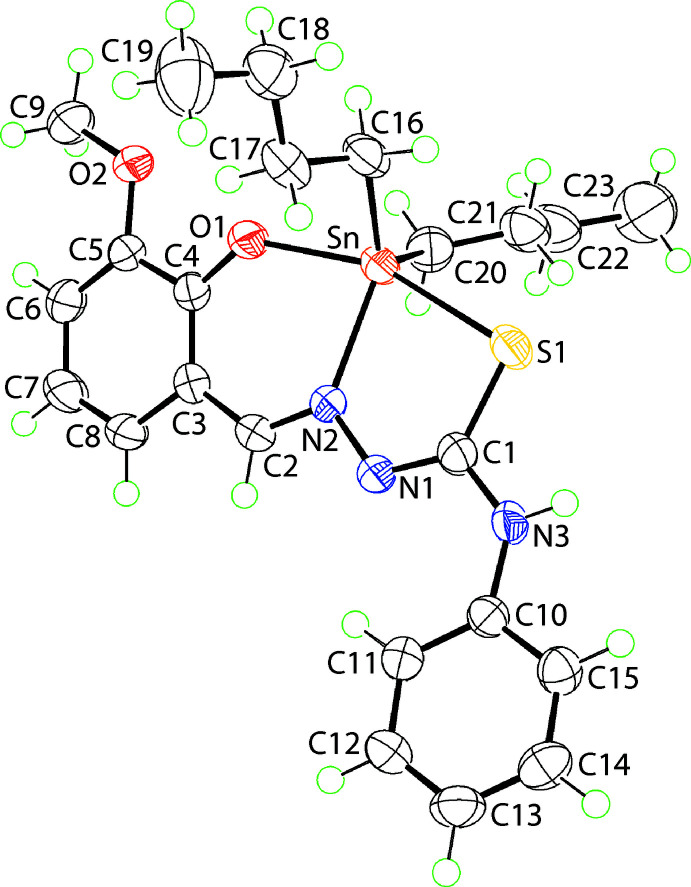
The mol­ecular structure of (I) showing the atom-labelling scheme and displacement ellipsoids at the 70% probability level.

The mol­ecular structure of (I), Sn(C_15_H_13_N_3_O_2_S)(C_4_H_9_)_2_ (Fig. 1 ▸), comprises a five-coordinate tin centre, being coordin­ated by a tridentate Schiff base di-anion and two *n*-butyl groups leading to a C_2_NOS donor set. Selected geometric parameters for (I) are collated in Table 1 ▸. While the direct acid analogue for the Schiff base in (I) has yet to be characterized crystallographically, the 4-meth­oxy analogue is known (Rubčić *et al.*, 2008 ▸). Compared to the S1—C1 [1.747 (3) Å], C1—N1 [1.304 (3) Å] and C2—N2 [1.311 (3) Å] bond lengths in (I), the equivalent bonds in the acid are 1.6769 (14), 1.3441 (17) and 1.2798 (18) Å, respectively (Spek, 2020 ▸), consistent with elongation, shortening and elongation in (I), respectively, confirming the presence of the thiol­ate-S1 and imine-N1 atoms. The angles subtended at the tin centre, Table 1 ▸, indicate a highly distorted coordination geometry. The angle closest to a *trans* angle is 157.56 (5)°, for S1—Sn—O1, with the next two widest angles being N2—Sn—C16 [126.42 (9)°] and C16—Sn—C20 [124.08 (11)°]. The distortion from the ideal square-pyramidal and trigonal-bipyramidal geometries is qu­anti­fied by the value of τ, with values of 0.0 and 1.0, respectively (Addison *et al.*, 1984 ▸). For (I), this computes to 0.52, being almost exactly between the two extreme values.

The N,O,S mode of coordination of the Schiff base di-anion gives rise to the formation of five- and six-membered chelate rings, the acute chelate angles, Table 1; these are partly responsible for the observed distortions in the coordination environment. The former ring, comprising the Sn, S1, N1, N2 and C1 atoms is almost planar, presenting a r.m.s. deviation of 0.0087 Å: atom N3 lies 0.016 (3) Å out of this plane. By contrast, distortions are evident in the six-membered chelate ring, defined by the Sn, O1, N2, C2–C4 atoms. The simplest description for the conformation is that of an envelope with the tin atom lying 0.519 (3) Å out of the plane defined by the remaining five atoms (r.m.s. deviation = 0.0379 Å). The dihedral angle between the five-membered chelate ring and the best plane through the five approximately co-planar atoms of the six-membered chelate ring is 13.59 (12)°, that between the five-membered and N-bound phenyl ring is 6.92 (12)° and that between the peripheral C_6_ rings is 19.63 (13)°, highlighting the observation the Schiff base di-anion deviates significantly from co-planarity.

## Supra­molecular features   

**Figure 2:**
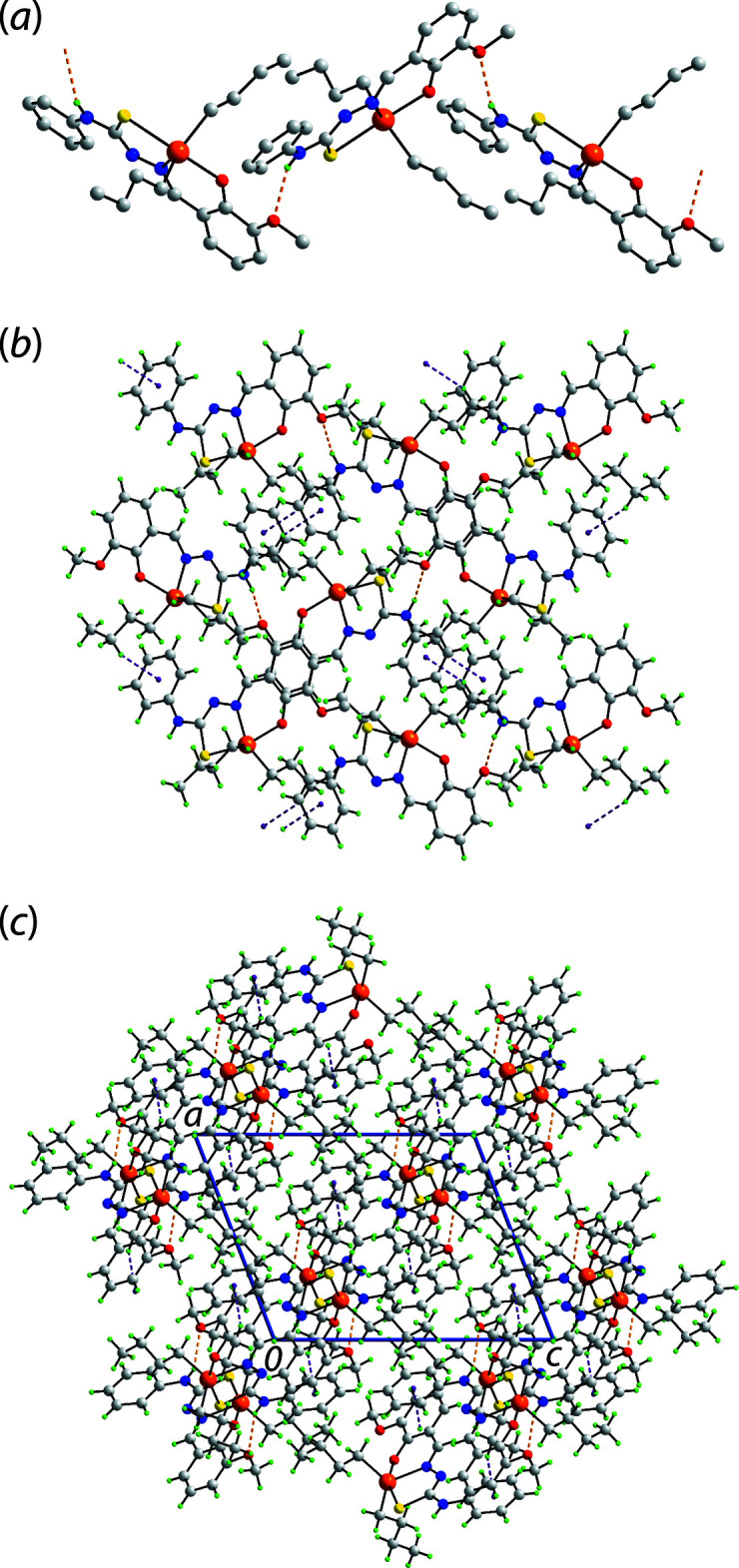
Mol­ecular packing in (I): (*a*) the helical, supra­molecular chain sustained by amine-*N*—*H*⋯*O*(meth­oxy) hydrogen bonding shown as orange dashed lines (non-participating H atoms omitted), (*b*) the supra­molecular layer parallel to (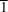
01) whereby the chains of (*a*) are connected by methyl­ene-C—H⋯π(phen­yl) inter­actions shown as purple dashed lines and (*c*) a view of the unit-cell contents shown in projection down the *b*-axis direction.

Conventional hydrogen bonding is noted in the crystal of (I), Table 2 ▸. Thus, amine-N—H⋯O(meth­oxy) hydrogen bonds assemble mol­ecules into a helical, supra­molecular chain propagating along the *b*-axis direction, Fig. 2 ▸(*a*). The only other directional inter­actions based on an analysis of the points of contact between mol­ecules in the crystal (Spek, 2020 ▸), are methyl­ene-C—H⋯π(phen­yl) inter­actions. These lead to a supra­molecular layer parallel to (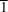
01), Fig. 2 ▸(*b*). Layers stack without specific inter­actions between them, Fig. 2 ▸(*c*).

## Analysis of the Hirshfeld surfaces   

**Figure 3:**
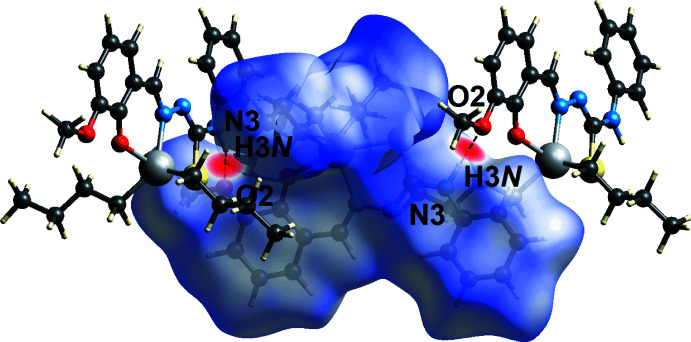
A view of the Hirshfeld surface for (I) mapped over *d*_norm_ in the range −0.40 to +1.61 arbitrary units, highlighting red spots due to N3—H3*N*⋯O2 hydrogen bonds.

**Figure 4:**
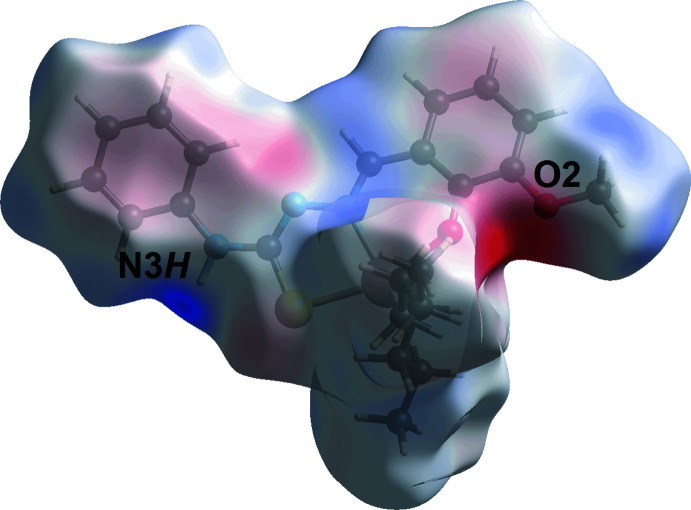
A view of the Hirshfeld surface mapped over the calculated electrostatic potential for (I) in the range −0.095 to 0.095 a.u.

**Figure 5:**
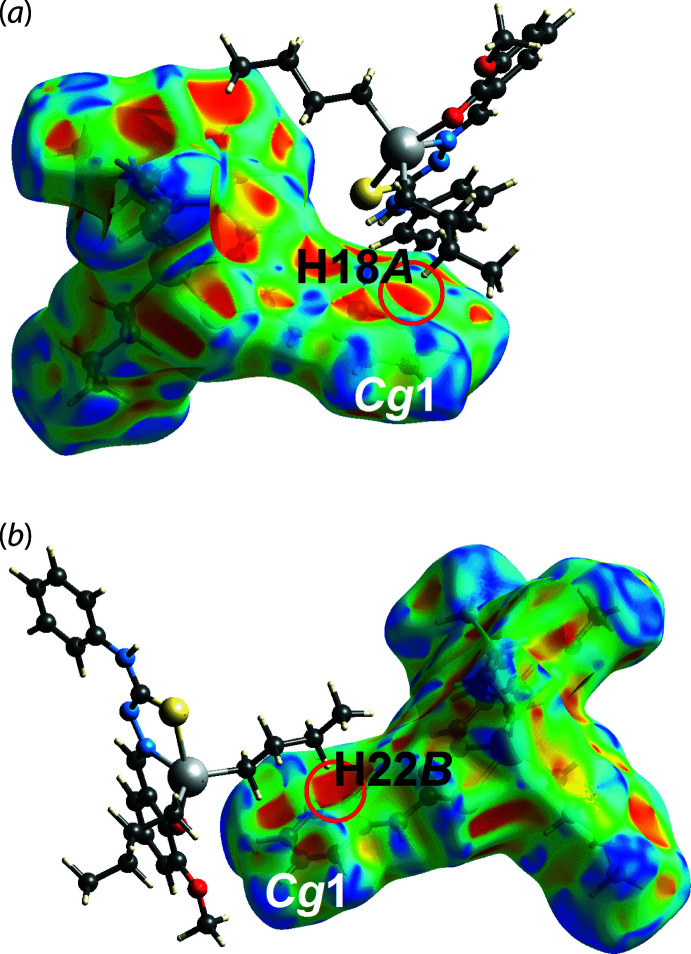
Views of the Hirshfeld surface for (I) mapped over the shape-index property. The influence of the (*a*) H18*A*⋯*Cg*1 and (*b*) H22*B*⋯*Cg*1 contacts are highlighted by the hollows emphasized by the red circles.

The Hirshfeld surface analysis for (I) was conducted to ascertain further information on the supra­molecular association between mol­ecules in the crystal, in particular in the inter-layer region. The calculated Hirshfeld surface was mapped over the normalized contact distance *d*_norm_ (McKinnon *et al.*, 2004 ▸) and electrostatic potential (Spackman *et al.*, 2008 ▸), and the associated two-dimensional fingerprint plots were calculated using *Crystal Explorer 17* (Turner *et al.*, 2017 ▸) following a literature procedure (Tan *et al.*, 2019 ▸). The electrostatic potentials were calculated using the STO-3G basis set at the Hartree–Fock level of theory. The only red spots observed on the Hirshfeld surface mapped over *d*_norm_, Fig. 3 ▸, arose as a result of the conventional amine-N3—H3*N*⋯O2(meth­oxy) hydrogen bond. This hydrogen bond is also reflected in the Hirshfeld surface mapped over the electrostatic potential, Fig. 4 ▸, where the positive electrostatic potential (blue) and negative electrostatic potential (red) regions are evident around the amine-H3*N* and meth­oxy-O2 atoms, respectively. Complementing the methyl­ene-C18—H18*A*⋯*Cg*1 contact listed in Table 2 ▸, is a longer methyl­ene-C22—H22*B*⋯*Cg*1 contact in the inter-layer region, Table 3 ▸. Each inter­action is observed as an orange ‘hollow’ on the Hirshfeld surface mapped over shape-index property, Fig. 5 ▸.

**Figure 6:**
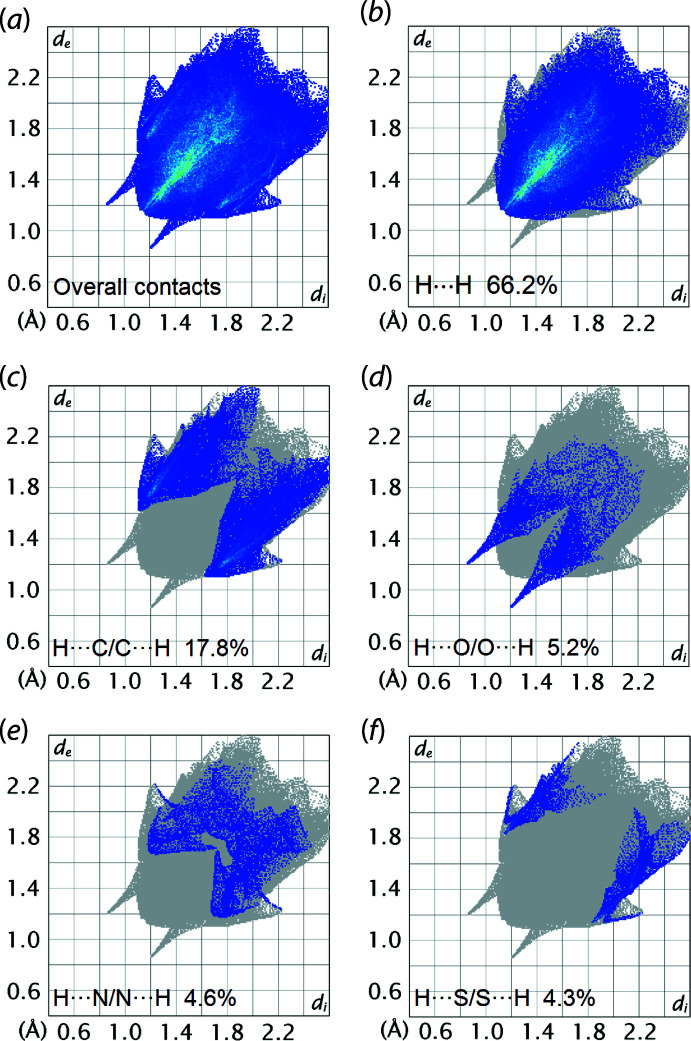
(*a*) A comparison of the full two-dimensional fingerprint plot for (I) and those delineated into (*b*) H⋯H, (*c*) H⋯C/C⋯H, (*d*) H⋯O/O⋯H, (*e*) H⋯N/N⋯H and (*f*) H⋯S/S⋯H contacts.

The overall two-dimensional fingerprint plot for the Hirshfeld surface of (I) is shown with characteristic pseudo-symmetric wings in the upper left and lower right sides of the *d*_e_ and *d*_i_ diagonal axes, respectively, in Fig. 6 ▸(*a*). The individual H⋯H, H⋯C/C⋯H, H⋯O/O⋯H, H⋯N/N⋯H and H⋯S/S⋯H contacts are illustrated in the delineated fingerprint plots in Fig. 6 ▸(*b*)–(*f*), respectively. The percentage contributions for the different inter­atomic contacts to the Hirshfeld surface are included in Fig. 6 ▸. The greatest contribution to the overall Hirshfeld surface is from H⋯H contacts, *i.e*. 66.2%. The H⋯H contacts appear as a beak-like distrib­ution tipped at *d*_e_ + *d*_i_ ∼2.4 Å in Fig. 6 ▸(*b*), with the short value corresponding to the H6⋯H22*A* and H7⋯H17*A* contacts, with details listed in Table 3 ▸. The H⋯C/C⋯H contacts contribute 17.8% and appear as two sharp-symmetric wings at *d*_e_ + *d*_i_ ∼2.7 Å, Fig. 6 ▸(*c*). This feature reflects the C—H⋯ π contacts as discussed above. Although H⋯O/O⋯H contacts only contribute 5.2% to the overall Hirshfeld surface, they appear as the shortest contacts at *d*_e_ + *d*_i_ ∼2.1 Å, being 0.6 Å shorter than the sum of their van der Waals radii, Fig. 6 ▸(*d*), and reflect the conventional hydrogen bonding leading to the supra­molecular chain. The H⋯N/N⋯H and H⋯S/S⋯H contacts contribute 4.6 and 4.3%, respectively, to the overall Hirshfeld surface. These contacts are reflected as pseudo-mirrored features at *d*_e_ + *d*_i_ ∼3.0 Å in each of Fig. 6 ▸(*e*) and (*f*), with the minimum distance being around the sum of their respective van der Waals radii. The other inter­atomic contacts, *i.e*. C⋯C and C⋯N/N⋯C, have a negligible effect on the mol­ecular packing and their contributions to the overall Hirshfeld surface are 1.7 and 0.2%, respectively.

## Computational chemistry   

In the present analysis, the pairwise inter­action energies between the mol­ecules in the crystal of (I) were calculated using the wave function at the B3LYP/DGDZVP level of theory. The total inter­action energies (*E*_tot_) as well as individual energy components, namely electrostatic (*E*_ele_), polarization (*E*_pol_), dispersion (*E*_dis_) and exchange-repulsion (*E*_rep_) are collated in Table 4 ▸. The most significant stabilization energies in the intra-layer region arise from the amine-N3—H3*N*⋯O2(meth­oxy) hydrogen bond (*E*_tot_ = −83.4 kJ mol^−1^). In addition to the methyl­ene-C18—H18*A*⋯*Cg*1 contacts, mol­ecules in the intra-layer region are stabilized by a number of H⋯H contacts, notably H7⋯H17*A* contacts with a separation of 2.32 Å, Table 3 ▸. Therefore, the dispersion term, *i.e. E*_dis_, makes the major contribution to the overall inter­action energy in the intra-layer region.

**Figure 7:**
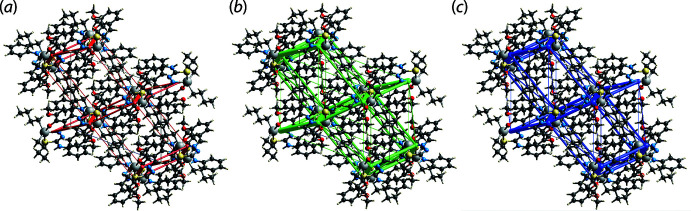
Perspective views of the energy frameworks calculated for (I) showing (*a*) electrostatic potential force, (*b*) dispersion force and (*c*) total energy, each plotted down the *b* axis. The radii of the cylinders are proportional to the relative magnitudes of the corresponding energies and were adjusted to the same scale factor of 55 with a cut-off value of 5 kJ mol^−1^.

The greatest stabilization energies in the inter-layer region relate to the weak methyl­ene-C22—H22*B*⋯*Cg*1 contact (*E*_tot_ = −25.7 kJ mol^−1^) with the remaining inter­molecular contacts between mol­ecules being stabilizing H⋯H contacts. The nature of these contacts leads to the dominance of the *E*_dis_ component in the mol­ecular packing, Table 4 ▸. This observation is also highlighted in the energy framework diagrams of Fig. 7 ▸, where the magnitudes of inter­molecular energies are represented graphically in the form of cylinders; the wider the cylinder, the greater the energy. The total *E*_ele_ and *E*_dis_ components of all pairwise inter­actions sum to −127.0 and −329.5 kJ mol^−1^, respectively.

## Database survey   

**Figure 8:**
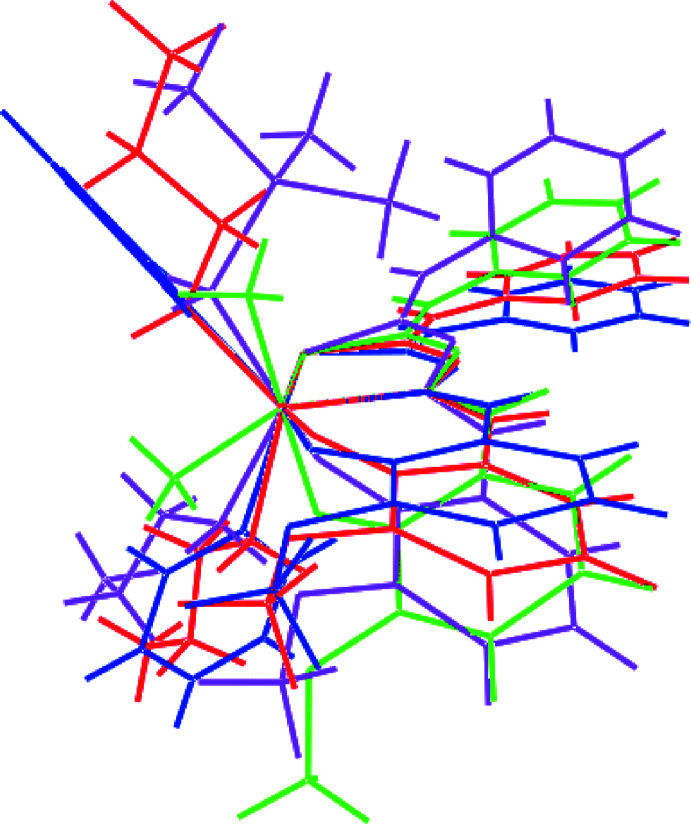
An overlay diagram of (I) red image, (II) green, (III) blue and (IV*a*) pink. The mol­ecules have been overlapped so that the Sn, S1 and N2 atoms of each mol­ecule are coincident.

The crystal structure determination of (I) represents the fourth example of a diorganotin derivative containing the same Schiff base ligand, *i.e. RR*’Sn(*L*). Each of the literature structures were reported during 2020, *i.e. R* = *R*′ = Me (II) and Ph (III) (Cambridge Structural Database refcodes MUWQED and MUWQAZ, respectively; Yusof *et al.*, 2020 ▸) and *R* = *n*-Bu and *R*′ = CH_2_SiMe_3_ (IV; CUJHIB; Xie *et al.*, 2020 ▸). It is noted that the *R* = *R*′ = Ph derivative (III) co-crystallized with one-half mole equivalent of 3-meth­oxy­salicyl­aldehyde azine (Yusof *et al.*, 2020 ▸). Also, two positions were modelled for the tin atom in (IV), with the major component having a site occupancy factor = 0.523 and is designated hereafter as (IV*a*). Selected geometric parameters for the four structures are collated in Table 5 ▸ and an overlay diagram for (I)–(IV*a*) is shown in Fig. 8 ▸. None of the mol­ecules has crystallographic symmetry and all present distorted C_2_NOS coordination geometries. With the exception of (II), the mol­ecules have inter­mediate coordination geometries with τ (Addison *et al.*, 1984 ▸) ranging from 0.52 in (I) to 0.60 in each of (III) and (IV*a*). The standout mol­ecule is the di­methyl­tin derivative (II) which, with τ = 0.00, is well described as having a square-pyramidal geometry. The S1—Sn—O1 angles span a range greater than 15°, *i.e*. 145.67 (9) in (II) to 161.81 (7)° in (III).

The hydrogen-bonding patterns formed in the crystals of (I)–(IV) are also distinct. Supra­molecular helical chains, sustained by amine-H⋯O(meth­oxy) hydrogen bonds are found in each of (I) and (IV). However, in (II), the inter­actions leading to a helical chain are of the type amine-H⋯O(phenoxide). A further distinction is noted in the crystal of (III) in that dimeric aggregates are formed, featuring amine-N—H⋯S(thiol­ate) hydrogen bonding.

## Synthesis and crystallization   

The synthesis of the Schiff base precursor, [2-(2-hy­droxy-3-meth­oxy­benzyl­idene)-*N*-phenyl­hydrazine carbo­thio­amide] was according to the procedure described in the literature (Đilović *et al.*, 2008 ▸; Kalaivani *et al.*, 2012 ▸) with some modifications (Yusof *et al.*, 2020 ▸). 4-Phenyl­thio­semicarbazide (1.67 g, 10 mmol) was dissolved in methanol (40 ml) with stirring and heating (313 K) over a period of 30 min. 2-Hy­droxy-3-meth­oxy­benzaldehyde (1.52 g, 10 mmol) in methanol (10 ml) was added to the thio­semicarbazide solution and stirred at room temperature for 4 h. Upon cooling, a crystalline product began to form which was filtered, washed with cold methanol and dried in a desiccator over anhydrous silica gel.

Synthesis of (I): The Schiff base (0.60 g, 2 mmol) was dissolved in a mixture of ethanol:DMF (7:3; 100 ml). Then, Et_3_N (0.28 ml, 2 mmol) was added dropwise followed by reflux for 2 h. Then, di­butyl­tin(IV) dichloride (0.61 g, 2 mmol) was added to the mixture followed by reflux for 6 h. The mixture was filtered while hot to remove the [Et_3_NH]Cl salt that formed and the filtrate was kept at room temperature until bright-yellow crystals appeared. Yield 62%, m.p. 384–385 K. FT–IR (ATR, cm^−1^): 3322 ν(N—H), 1582 ν(C=N), 1076 ν(N—N), 853 ν(C=S). ^1^H NMR (CDCl_3_, 700 MHz) δ: 8.63 (*s*, 1H, NH), 7.54 (*s*, 1H, N—CH), 6.65–7.32 (*m*, 8H, Ar—H), 3.85 (*s*, 3H, O—CH_3_), *n*-Bu: 1.68 [*t*, 4H, Hα], 1.61 [*m*, 4H, Hβ], 1.34 [*m*, 4H, Hγ], 0.87 [*t*, 6H, Hδ]. ^13^C NMR (CDCl_3_, 175 MHz) δ: 162.5 (S_2_C), 159.0 (C=N), 151.3, 139.6, 128.9, 125.4, 123.1, 120.4, 120.0, 116.9, 116.3, 115.7 (Ar—C), 56.3 (O—CH_3_), *n*-Bu: 27.5 (Cα), 26.5 (Cβ), 26.0 (Cγ), 13.6 (Cδ).

## Refinement   

Crystal data, data collection and structure refinement details are summarized in Table 6 ▸. The carbon-bound H atoms were placed in calculated positions (C—H = 0.95–0.99 Å) and were included in the refinement in the riding-model approximation, with *U*_iso_(H) set to 1.2–1.5*U*_eq_(C). The N-bound H atom was located in a difference-Fourier map, but was refined with a N—H = 0.88±0.01 Å distance restraint, and with *U*_iso_(H) set to 1.2*U*_eq_(N). The maximum and minimum residual electron density peaks of 1.63 and 0.52 e Å^−3^, respectively, were located 0.97 and 0.53 Å from the Sn atom.

## Supplementary Material

Crystal structure: contains datablock(s) I, global. DOI: 10.1107/S2056989021001870/hb7969sup1.cif

Structure factors: contains datablock(s) I. DOI: 10.1107/S2056989021001870/hb7969Isup2.hkl

CCDC reference: 2063179

Additional supporting information:  crystallographic information; 3D view; checkCIF report

## Figures and Tables

**Table 1:** Selected geometric parameters (Å, °)

Sn—S1	2.5598 (7)	Sn—C20	2.140 (3)
Sn—O1	2.1089 (16)	S1—C1	1.747 (3)
Sn—N2	2.212 (2)	N1—C1	1.304 (3)
Sn—C16	2.136 (3)	N2—C2	1.311 (3)
			
S1—Sn—O1	157.56 (5)	O1—Sn—C16	88.11 (8)
S1—Sn—N2	77.04 (5)	O1—Sn—C20	93.08 (9)
S1—Sn—C16	96.03 (8)	N2—Sn—C16	126.42 (9)
S1—Sn—C20	102.61 (8)	N2—Sn—C20	109.11 (10)
O1—Sn—N2	82.75 (7)	C16—Sn—C20	124.08 (11)

**Table 2:** Hydrogen-bond geometry (Å, °) *Cg*1 is the centroid of the (C10–C15) ring.

*D*—H⋯*A*	*D*—H	H⋯*A*	*D*⋯*A*	*D*—H⋯*A*
N3—H3*N*⋯O2^i^	0.87 (2)	2.21 (2)	2.990 (3)	150 (2)
C18—H18*A*⋯*Cg*1^ii^	0.99	2.81	3.730 (3)	154

Symmetry codes: (i) -x+{\script{1\over 2}}, y+{\script{1\over 2}}, -z+{\script{1\over 2}}; (ii) x-{\script{1\over 2}}, -y+{\script{3\over 2}}, z-{\script{1\over 2}}.

**Table 3:** A summary of short inter­atomic contacts (Å) for (I)*^*a*^*

Contact	Distance	Symmetry operation
N3—H3*N*⋯O2	2.09	−*x* + {1\over 2}, *y* + {1\over 2}, −*z* + {1\over 2}
C18—H18*A*⋯*Cg*1	2.81	*x* − {1\over 2}, −*y* − {3\over 2}, *z* − {1\over 2}
C22—H22*B*⋯*Cg*1	3.28	*x* + {1\over 2}, −*y* + {3\over 2}, *z* − {1\over 2}
H6⋯H22*A*	2.32	−*x* + {3\over 2}, *y* − {1\over 2}, −*z* + {1\over 2}
H7⋯H17*A*	2.32	−*x* + 1, −*y* + 1, −*z* + 1

Note: (*a*) The inter­atomic distances are calculated in *Crystal Explorer 17* (Turner *et al.*, 2017 ▸) with the *X*—H bond lengths adjusted to their neutron values.

**Table 4:** A summary of inter­action energies (kJ mol^−1^) calculated for (I)

Contact	*R* (Å)	*E*_ele_	*E*_pol_	*E*_dis_	*E*_rep_	*E*_tot_
Intra-layer region						
N3—H3*N*⋯O2^i^	8.36	−51.6	−10.0	−77.3	74.2	−83.4
H7⋯H17*A* ^iv^ +						
H9*C*⋯H11^iv^ +						
H9*C*⋯H12^iv^	7.54	−30.2	−3.3	−88.0	78.5	−62.5
C18—H18*A*⋯*Cg*1^ii^+						
H8⋯H21*A* ^v^	7.93	−19.5	−2.6	−49.7	43.0	−39.2
H16*A*⋯H18*B* ^vi^ +						
H16*B*⋯H18*A* ^vi^	10.52	−4.3	−0.1	−24.5	18.6	−14.5
						
Inter-layer region						
C22—H22*B*⋯*Cg*1^iii^	11.00	−6.8	−1.3	−32.0	16.7	−25.7
H9*A*⋯H22*B* ^vii^+						
H9*A*⋯H23*A* ^vii^	11.46	−2.9	−0.7	−18.7	12.0	−12.6
H14⋯H15^viii^	15.83	−6.7	−0.7	−14.1	18.3	−8.6
H6⋯H22*A* ^ix^+						
H7⋯H23*B* ^ix^	12.63	−1.5	−0.4	−11.6	6.0	−8.3
H9*A*⋯H19*C* ^*x*^	11.27	−1.3	−0.2	−6.4	3.1	−5.2
H12⋯H23*A* ^xi^	14.28	−2.2	−0.1	−7.2	6.1	−4.9

Symmetry operations: (i) −*x* + {1\over 2}, *y* + {1\over 2}, −*z* + {1\over 2}; (ii) *x* − {1\over 2}, −*y* + {3\over 2}, *z* − {1\over 2}; (iii) *x* + {1\over 2}, −*y* + {3\over 2}, *z* − {1\over 2}; (iv) −*x* + 1, −*y* + 1, −*z* + 1; (v) *x* + {1\over 2}, −*y* + {3\over 2}, *z* + {1\over 2}; (vi) −*x*, −*y* + 1, −*z*; (vii) −*x* + 1, −*y* + 1, −*z*; (viii) −*x*, −*y* + 2, −*z* + 1; (ix) −*x* + {3\over 2}, *y* − {1\over 2}, −*z* + {1\over 2}; (*x*) *x* + 1, *y*, *z*; (xi) *x*, *y*, *z* + 1.

**Table 5:** A comparison of key geometric parameters (Å, °) in structures related to (I)

Compound	*R*, *R*′	Sn—S	Sn—O1	Sn—N2	S1—Sn—O1	C—Sn—C	τ	Ref.
(I)	*n*Bu, *n*Bu	2.5598 (7)	2.1089 (16)	2.212 (2)	157.56 (5)	124.08 (11)	0.52	This work
(II)	Me, Me	2.4982 (12)	2.085 (3)	2.257 (3)	145.67 (9)	114.82 (18)	0.00	Yusof *et al.* (2020 ▸)
(III)	Ph, Ph	2.5475 (8)	2.0853 (19)	2.176 (3)	161.81 (7)	121.46 (12)	0.60	Yusof *et al.* (2020 ▸)
(IV)*^*a*^*	*n*Bu, CH_2_Si*M*_3_	2.485 (5)	2.152 (6)	2.184 (6)	159.4 (2)	121.6 (4)	0.60	Xie *et al.* (2020 ▸)
(IV)*^*b*^*		2.587 (4)	2.063 (6)	2.218 (7)	157.6 (3)	123.8 (4)	0.56	

Notes: (*a*) major component of the disorder with a site occupancy = 0.527 and (*b*) minor component with occupancy 0.473.

**Table 6:** Experimental details

Crystal data	
Chemical formula	[Sn(C_4_H_9_)_2_(C_15_H_13_N_3_O_2_S)]
*M*_r_	532.26
Crystal system, space group	Monoclinic, *P*2_1_/*n*
Temperature (K)	150
*a*, *b*, *c* (Å)	11.2720 (3), 16.1954 (3), 14.2778 (3)
β (°)	111.180 (3)
*V* (Å^3^)	2430.41 (10)
*Z*	4
Radiation type	Mo *K*α
μ (mm^−1^)	1.16
Crystal size (mm)	0.15 × 0.10 × 0.06

Data collection	
Diffractometer	Rigaku Oxford Diffraction Xcalibur, Eos, Gemini
Absorption correction	Multi-scan (*CrysAlis PRO*; Rigaku OD, 2015 ▸)
*T*_min_, *T*_max_	0.850, 1.000
No. of measured, independent and observed [*I* > 2σ(*I*)] reflections	26999, 5967, 4699
*R*_int_	0.051
(sin θ/λ)_max_ (Å^−1^)	0.689

Refinement	
*R*[*F* ^2^ > 2σ(*F* ^2^)], *wR*(*F* ^2^), *S*	0.033, 0.076, 1.04
No. of reflections	5967
No. of parameters	277
No. of restraints	1
H-atom treatment	H atoms treated by a mixture of independent and constrained refinement
Δρ_max_, Δρ_min_ (e Å^−3^)	1.63, −0.52

Computer programs: *CrysAlis PRO* (Rigaku OD, 2015 ▸), *SHELXT* (Sheldrick, 2015*a* ▸), *SHELXL2018/3* (Sheldrick, 2015*b* ▸), *ORTEP-3 for Windows* (Farrugia, 2012 ▸), *DIAMOND* (Brandenburg, 2006 ▸) and *publCIF* (Westrip, 2010 ▸).

## supplementary crystallographic information

### Crystal data

**Table:**

[Sn(C_4_H_9_)_2_(C_15_H_13_N_3_O_2_S)]	*F*(000) = 1088
*M**_r_* = 532.26	*D*_x_ = 1.455 Mg m^−^^3^
Monoclinic, *P*2_1_/*n*	Mo *K*α radiation, λ = 0.71073 Å
*a* = 11.2720 (3) Å	Cell parameters from 9470 reflections
*b* = 16.1954 (3) Å	θ = 3.8–28.9°
*c* = 14.2778 (3) Å	µ = 1.16 mm^−^^1^
β = 111.180 (3)°	*T* = 150 K
*V* = 2430.41 (10) Å^3^	Prism, yellow
*Z* = 4	0.15 × 0.10 × 0.06 mm

### Data collection

**Table:**

Rigaku Oxford Diffraction Xcalibur, Eos, Gemini diffractometer	5967 independent reflections
Radiation source: fine-focus sealed X-ray tube, Enhance (Mo) X-ray Source	4699 reflections with *I* > 2σ(*I*)
Graphite monochromator	*R*_int_ = 0.051
Detector resolution: 16.1952 pixels mm^-1^	θ_max_ = 29.3°, θ_min_ = 3.8°
ω scans	*h* = −15→14
Absorption correction: multi-scan (CrysAlis PRO; Rigaku OD, 2015)	*k* = −21→22
*T*_min_ = 0.850, *T*_max_ = 1.000	*l* = −19→18
26999 measured reflections	

### Refinement

**Table:**

Refinement on *F*^2^	Primary atom site location: structure-invariant direct methods
Least-squares matrix: full	Secondary atom site location: difference Fourier map
*R*[*F*^2^ > 2σ(*F*^2^)] = 0.033	Hydrogen site location: mixed
*wR*(*F*^2^) = 0.076	H atoms treated by a mixture of independent and constrained refinement
*S* = 1.03	*w* = 1/[σ^2^(*F*_o_^2^) + (0.0297*P*)^2^ + 1.1545*P*] where *P* = (*F*_o_^2^ + 2*F*_c_^2^)/3
5967 reflections	(Δ/σ)_max_ = 0.001
277 parameters	Δρ_max_ = 1.63 e Å^−^^3^
1 restraint	Δρ_min_ = −0.52 e Å^−^^3^

### Special details

**Table:**

Geometry. All esds (except the esd in the dihedral angle between two l.s. planes) are estimated using the full covariance matrix. The cell esds are taken into account individually in the estimation of esds in distances, angles and torsion angles; correlations between esds in cell parameters are only used when they are defined by crystal symmetry. An approximate (isotropic) treatment of cell esds is used for estimating esds involving l.s. planes.

### Fractional atomic coordinates and isotropic or equivalent isotropic displacement parameters (Å^2^)

**Table:**

	*x*	*y*	*z*	*U*_iso_*/*U*_eq_	
Sn	0.30782 (2)	0.63922 (2)	0.20843 (2)	0.02658 (7)	
S1	0.17922 (7)	0.76839 (5)	0.21316 (5)	0.03737 (18)	
O1	0.41536 (16)	0.53123 (10)	0.26242 (13)	0.0299 (4)	
O2	0.55992 (16)	0.40842 (10)	0.25269 (13)	0.0279 (4)	
N1	0.3367 (2)	0.74015 (12)	0.40760 (15)	0.0271 (5)	
N2	0.38235 (19)	0.67314 (12)	0.36939 (15)	0.0245 (5)	
N3	0.1999 (2)	0.85096 (13)	0.37649 (16)	0.0278 (5)	
H3N	0.142 (2)	0.8768 (15)	0.3282 (15)	0.033*	
C1	0.2476 (2)	0.78406 (15)	0.34279 (18)	0.0267 (6)	
C2	0.4807 (2)	0.63876 (15)	0.43827 (19)	0.0266 (5)	
H2	0.507201	0.663195	0.503089	0.032*	
C3	0.5533 (2)	0.56903 (15)	0.42817 (18)	0.0253 (5)	
C4	0.5195 (2)	0.51976 (14)	0.34156 (18)	0.0231 (5)	
C5	0.5993 (2)	0.45193 (15)	0.34154 (18)	0.0240 (5)	
C6	0.7057 (2)	0.43422 (16)	0.4235 (2)	0.0315 (6)	
H6	0.758465	0.388936	0.421749	0.038*	
C7	0.7367 (3)	0.48344 (17)	0.5104 (2)	0.0373 (7)	
H7	0.809490	0.470558	0.567769	0.045*	
C8	0.6629 (2)	0.54928 (16)	0.51260 (19)	0.0320 (6)	
H8	0.685130	0.582370	0.571384	0.038*	
C9	0.6339 (3)	0.33893 (18)	0.2478 (2)	0.0414 (7)	
H9A	0.720214	0.356915	0.256299	0.062*	
H9B	0.595020	0.311870	0.182395	0.062*	
H9C	0.637687	0.299962	0.301309	0.062*	
C10	0.2229 (2)	0.87931 (15)	0.47493 (19)	0.0246 (5)	
C11	0.3071 (3)	0.84289 (16)	0.5628 (2)	0.0321 (6)	
H11	0.356486	0.796350	0.559100	0.039*	
C12	0.3174 (3)	0.87544 (17)	0.6549 (2)	0.0355 (7)	
H12	0.374328	0.850489	0.714380	0.043*	
C13	0.2475 (3)	0.94306 (17)	0.6629 (2)	0.0353 (6)	
H13	0.254424	0.963702	0.726972	0.042*	
C14	0.1671 (3)	0.98029 (17)	0.5762 (2)	0.0331 (6)	
H14	0.119945	1.027809	0.580593	0.040*	
C15	0.1547 (2)	0.94894 (16)	0.4831 (2)	0.0286 (6)	
H15	0.099062	0.975153	0.424064	0.034*	
C16	0.1475 (2)	0.56202 (17)	0.13464 (19)	0.0320 (6)	
H16A	0.073373	0.597705	0.099569	0.038*	
H16B	0.165615	0.529157	0.082891	0.038*	
C17	0.1111 (3)	0.50319 (18)	0.2027 (2)	0.0368 (7)	
H17A	0.078865	0.535372	0.247468	0.044*	
H17B	0.187726	0.472697	0.245367	0.044*	
C18	0.0095 (3)	0.44156 (18)	0.1427 (2)	0.0408 (7)	
H18A	−0.067397	0.472273	0.101010	0.049*	
H18B	0.041312	0.410471	0.096902	0.049*	
C19	−0.0267 (3)	0.3812 (2)	0.2085 (3)	0.0630 (11)	
H19A	0.046823	0.346452	0.244721	0.095*	
H19B	−0.096854	0.346334	0.166641	0.095*	
H19C	−0.053386	0.411610	0.256866	0.095*	
C20	0.4355 (3)	0.68370 (17)	0.1396 (2)	0.0348 (6)	
H20A	0.510109	0.708354	0.192719	0.042*	
H20B	0.466511	0.635693	0.112094	0.042*	
C21	0.3833 (3)	0.74679 (18)	0.0563 (2)	0.0385 (7)	
H21A	0.345417	0.793150	0.081061	0.046*	
H21B	0.314902	0.720923	−0.000729	0.046*	
C22	0.4850 (3)	0.7805 (2)	0.0194 (2)	0.0487 (8)	
H22A	0.555087	0.804035	0.077308	0.058*	
H22B	0.520427	0.734251	−0.007521	0.058*	
C23	0.4367 (5)	0.8464 (2)	−0.0612 (3)	0.0684 (11)	
H23A	0.360725	0.826100	−0.115308	0.103*	
H23B	0.502935	0.859307	−0.088338	0.103*	
H23C	0.415227	0.896385	−0.031958	0.103*	

### Atomic displacement parameters (Å^2^)

**Table:**

	*U*^11^	*U*^22^	*U*^33^	*U*^12^	*U*^13^	*U*^23^
Sn	0.02994 (10)	0.02799 (11)	0.01860 (10)	0.00565 (7)	0.00492 (7)	−0.00006 (7)
S1	0.0463 (4)	0.0399 (4)	0.0204 (3)	0.0194 (3)	0.0054 (3)	0.0010 (3)
O1	0.0297 (9)	0.0269 (9)	0.0246 (9)	0.0067 (7)	−0.0003 (7)	−0.0032 (7)
O2	0.0323 (9)	0.0233 (9)	0.0253 (9)	0.0031 (7)	0.0069 (8)	−0.0037 (7)
N1	0.0342 (12)	0.0242 (11)	0.0205 (11)	0.0081 (9)	0.0068 (9)	−0.0008 (9)
N2	0.0308 (11)	0.0213 (10)	0.0201 (11)	0.0058 (9)	0.0076 (9)	−0.0001 (8)
N3	0.0339 (12)	0.0270 (12)	0.0213 (11)	0.0122 (9)	0.0086 (9)	0.0040 (9)
C1	0.0315 (13)	0.0262 (13)	0.0229 (13)	0.0041 (11)	0.0104 (11)	0.0024 (10)
C2	0.0313 (13)	0.0267 (13)	0.0191 (12)	0.0018 (11)	0.0059 (10)	−0.0007 (10)
C3	0.0277 (12)	0.0239 (13)	0.0221 (13)	0.0028 (10)	0.0065 (10)	0.0027 (10)
C4	0.0235 (12)	0.0219 (12)	0.0223 (13)	0.0006 (10)	0.0062 (10)	0.0014 (10)
C5	0.0275 (12)	0.0211 (12)	0.0225 (13)	−0.0022 (10)	0.0079 (10)	−0.0009 (10)
C6	0.0323 (14)	0.0263 (14)	0.0337 (15)	0.0081 (11)	0.0093 (12)	0.0002 (11)
C7	0.0353 (15)	0.0399 (16)	0.0259 (15)	0.0094 (12)	−0.0019 (12)	−0.0005 (12)
C8	0.0353 (14)	0.0299 (14)	0.0224 (13)	0.0051 (11)	0.0004 (11)	−0.0045 (11)
C9	0.0522 (18)	0.0321 (15)	0.0359 (17)	0.0140 (13)	0.0109 (14)	−0.0065 (13)
C10	0.0283 (12)	0.0249 (13)	0.0225 (13)	0.0001 (10)	0.0114 (10)	0.0011 (10)
C11	0.0428 (16)	0.0264 (14)	0.0266 (14)	0.0062 (11)	0.0119 (12)	0.0015 (11)
C12	0.0498 (17)	0.0332 (15)	0.0219 (14)	0.0005 (13)	0.0108 (12)	0.0007 (11)
C13	0.0468 (17)	0.0341 (15)	0.0292 (15)	−0.0028 (13)	0.0187 (13)	−0.0071 (12)
C14	0.0334 (14)	0.0320 (14)	0.0391 (16)	0.0016 (12)	0.0193 (13)	−0.0049 (12)
C15	0.0268 (13)	0.0303 (14)	0.0295 (14)	0.0034 (11)	0.0111 (11)	0.0024 (11)
C16	0.0296 (13)	0.0392 (15)	0.0215 (13)	0.0030 (12)	0.0024 (11)	0.0001 (12)
C17	0.0366 (15)	0.0454 (17)	0.0230 (14)	0.0027 (13)	0.0041 (11)	0.0043 (12)
C18	0.0341 (15)	0.0447 (17)	0.0377 (17)	0.0015 (13)	0.0059 (13)	0.0018 (14)
C19	0.048 (2)	0.059 (2)	0.071 (3)	−0.0058 (17)	0.0099 (19)	0.0203 (19)
C20	0.0392 (15)	0.0353 (15)	0.0329 (16)	0.0039 (12)	0.0168 (13)	−0.0008 (12)
C21	0.0435 (16)	0.0439 (17)	0.0300 (15)	0.0005 (13)	0.0155 (13)	−0.0009 (13)
C22	0.059 (2)	0.056 (2)	0.0358 (17)	−0.0094 (17)	0.0237 (16)	−0.0045 (15)
C23	0.106 (3)	0.060 (2)	0.055 (2)	−0.008 (2)	0.048 (2)	0.0049 (19)

### Geometric parameters (Å, º)

**Table:**

Sn—S1	2.5598 (7)	C11—H11	0.9500
Sn—O1	2.1089 (16)	C12—C13	1.378 (4)
Sn—N2	2.212 (2)	C12—H12	0.9500
Sn—C16	2.136 (3)	C13—C14	1.381 (4)
Sn—C20	2.140 (3)	C13—H13	0.9500
S1—C1	1.747 (3)	C14—C15	1.382 (4)
O1—C4	1.316 (3)	C14—H14	0.9500
O2—C5	1.377 (3)	C15—H15	0.9500
O2—C9	1.417 (3)	C16—C17	1.519 (4)
N1—C1	1.304 (3)	C16—H16A	0.9900
N1—N2	1.394 (3)	C16—H16B	0.9900
N2—C2	1.311 (3)	C17—C18	1.528 (4)
N3—C1	1.371 (3)	C17—H17A	0.9900
N3—C10	1.411 (3)	C17—H17B	0.9900
N3—H3N	0.866 (10)	C18—C19	1.510 (4)
C2—C3	1.432 (3)	C18—H18A	0.9900
C2—H2	0.9500	C18—H18B	0.9900
C3—C4	1.404 (3)	C19—H19A	0.9800
C3—C8	1.416 (3)	C19—H19B	0.9800
C4—C5	1.420 (3)	C19—H19C	0.9800
C5—C6	1.370 (3)	C20—C21	1.516 (4)
C6—C7	1.408 (4)	C20—H20A	0.9900
C6—H6	0.9500	C20—H20B	0.9900
C7—C8	1.359 (4)	C21—C22	1.525 (4)
C7—H7	0.9500	C21—H21A	0.9900
C8—H8	0.9500	C21—H21B	0.9900
C9—H9A	0.9800	C22—C23	1.519 (5)
C9—H9B	0.9800	C22—H22A	0.9900
C9—H9C	0.9800	C22—H22B	0.9900
C10—C15	1.393 (3)	C23—H23A	0.9800
C10—C11	1.400 (4)	C23—H23B	0.9800
C11—C12	1.382 (4)	C23—H23C	0.9800
			
S1—Sn—O1	157.56 (5)	C11—C12—H12	119.1
S1—Sn—N2	77.04 (5)	C12—C13—C14	118.8 (3)
S1—Sn—C16	96.03 (8)	C12—C13—H13	120.6
S1—Sn—C20	102.61 (8)	C14—C13—H13	120.6
O1—Sn—N2	82.75 (7)	C13—C14—C15	120.5 (3)
O1—Sn—C16	88.11 (8)	C13—C14—H14	119.8
O1—Sn—C20	93.08 (9)	C15—C14—H14	119.8
N2—Sn—C16	126.42 (9)	C14—C15—C10	120.7 (2)
N2—Sn—C20	109.11 (10)	C14—C15—H15	119.6
C16—Sn—C20	124.08 (11)	C10—C15—H15	119.6
C1—S1—Sn	96.29 (8)	C17—C16—Sn	115.28 (17)
C4—O1—Sn	130.37 (15)	C17—C16—H16A	108.5
C5—O2—C9	116.9 (2)	Sn—C16—H16A	108.5
C1—N1—N2	116.5 (2)	C17—C16—H16B	108.5
C2—N2—N1	111.6 (2)	Sn—C16—H16B	108.5
C2—N2—Sn	125.07 (17)	H16A—C16—H16B	107.5
N1—N2—Sn	123.00 (14)	C16—C17—C18	111.8 (2)
C1—N3—C10	130.7 (2)	C16—C17—H17A	109.2
C1—N3—H3N	112.2 (19)	C18—C17—H17A	109.2
C10—N3—H3N	116.8 (19)	C16—C17—H17B	109.2
N1—C1—N3	118.8 (2)	C18—C17—H17B	109.2
N1—C1—S1	127.2 (2)	H17A—C17—H17B	107.9
N3—C1—S1	114.06 (17)	C19—C18—C17	113.0 (3)
N2—C2—C3	128.2 (2)	C19—C18—H18A	109.0
N2—C2—H2	115.9	C17—C18—H18A	109.0
C3—C2—H2	115.9	C19—C18—H18B	109.0
C4—C3—C8	119.8 (2)	C17—C18—H18B	109.0
C4—C3—C2	123.6 (2)	H18A—C18—H18B	107.8
C8—C3—C2	116.5 (2)	C18—C19—H19A	109.5
O1—C4—C3	123.4 (2)	C18—C19—H19B	109.5
O1—C4—C5	118.6 (2)	H19A—C19—H19B	109.5
C3—C4—C5	118.0 (2)	C18—C19—H19C	109.5
C6—C5—O2	124.8 (2)	H19A—C19—H19C	109.5
C6—C5—C4	121.4 (2)	H19B—C19—H19C	109.5
O2—C5—C4	113.7 (2)	C21—C20—Sn	116.88 (19)
C5—C6—C7	119.7 (2)	C21—C20—H20A	108.1
C5—C6—H6	120.1	Sn—C20—H20A	108.1
C7—C6—H6	120.1	C21—C20—H20B	108.1
C8—C7—C6	120.4 (2)	Sn—C20—H20B	108.1
C8—C7—H7	119.8	H20A—C20—H20B	107.3
C6—C7—H7	119.8	C20—C21—C22	112.7 (3)
C7—C8—C3	120.7 (2)	C20—C21—H21A	109.1
C7—C8—H8	119.7	C22—C21—H21A	109.1
C3—C8—H8	119.7	C20—C21—H21B	109.1
O2—C9—H9A	109.5	C22—C21—H21B	109.1
O2—C9—H9B	109.5	H21A—C21—H21B	107.8
H9A—C9—H9B	109.5	C23—C22—C21	113.9 (3)
O2—C9—H9C	109.5	C23—C22—H22A	108.8
H9A—C9—H9C	109.5	C21—C22—H22A	108.8
H9B—C9—H9C	109.5	C23—C22—H22B	108.8
C15—C10—C11	118.8 (2)	C21—C22—H22B	108.8
C15—C10—N3	116.0 (2)	H22A—C22—H22B	107.7
C11—C10—N3	125.2 (2)	C22—C23—H23A	109.5
C12—C11—C10	119.2 (3)	C22—C23—H23B	109.5
C12—C11—H11	120.4	H23A—C23—H23B	109.5
C10—C11—H11	120.4	C22—C23—H23C	109.5
C13—C12—C11	121.9 (3)	H23A—C23—H23C	109.5
C13—C12—H12	119.1	H23B—C23—H23C	109.5
			
C1—N1—N2—C2	173.0 (2)	O1—C4—C5—O2	4.0 (3)
C1—N1—N2—Sn	−1.2 (3)	C3—C4—C5—O2	−178.7 (2)
N2—N1—C1—N3	−179.6 (2)	O2—C5—C6—C7	179.8 (2)
N2—N1—C1—S1	−0.2 (4)	C4—C5—C6—C7	0.7 (4)
C10—N3—C1—N1	−6.2 (4)	C5—C6—C7—C8	−1.3 (4)
C10—N3—C1—S1	174.3 (2)	C6—C7—C8—C3	0.6 (5)
Sn—S1—C1—N1	1.1 (3)	C4—C3—C8—C7	0.7 (4)
Sn—S1—C1—N3	−179.43 (18)	C2—C3—C8—C7	179.1 (3)
N1—N2—C2—C3	180.0 (2)	C1—N3—C10—C15	−177.9 (3)
Sn—N2—C2—C3	−6.0 (4)	C1—N3—C10—C11	1.8 (4)
N2—C2—C3—C4	−7.4 (4)	C15—C10—C11—C12	1.8 (4)
N2—C2—C3—C8	174.2 (3)	N3—C10—C11—C12	−177.9 (3)
Sn—O1—C4—C3	26.6 (4)	C10—C11—C12—C13	−0.2 (4)
Sn—O1—C4—C5	−156.24 (18)	C11—C12—C13—C14	−1.5 (4)
C8—C3—C4—O1	175.9 (2)	C12—C13—C14—C15	1.6 (4)
C2—C3—C4—O1	−2.4 (4)	C13—C14—C15—C10	0.0 (4)
C8—C3—C4—C5	−1.2 (4)	C11—C10—C15—C14	−1.7 (4)
C2—C3—C4—C5	−179.5 (2)	N3—C10—C15—C14	178.0 (2)
C9—O2—C5—C6	1.9 (4)	Sn—C16—C17—C18	−171.27 (19)
C9—O2—C5—C4	−179.0 (2)	C16—C17—C18—C19	178.9 (3)
O1—C4—C5—C6	−176.8 (2)	Sn—C20—C21—C22	174.5 (2)
C3—C4—C5—C6	0.5 (4)	C20—C21—C22—C23	−177.7 (3)

### Hydrogen-bond geometry (Å, º)

*Cg*1 is the centroid of the (C10–C15) ring.

**Table:**

*D*—H···*A*	*D*—H	H···*A*	*D*···*A*	*D*—H···*A*
N3—H3*N*···O2^i^	0.87 (2)	2.21 (2)	2.990 (3)	150 (2)
C18—H18*A*···*Cg*1^ii^	0.99	2.81	3.730 (3)	154

Symmetry codes: (i) −*x*+1/2, *y*+1/2, −*z*+1/2; (ii) *x*−1/2, −*y*+3/2, *z*−1/2.
